# Unexpected Fetal Supraventricular Tachycardia Temporally Associated With Maternal Antihistamine Use: A Case Report

**DOI:** 10.1155/crog/6498180

**Published:** 2026-05-04

**Authors:** Khadijeh Riazi Kermani, Shahrokh Rajaei, Razieh Moazami Goudarzi, Nazanin Abdi

**Affiliations:** ^1^ Department of Pediatric Cardiology, Clinical Research Development Center of Children′s Hospital, Hormozgan University of Medical Sciences, Bandar Abbas, Hormozgan, Iran, hums.ac.ir; ^2^ Department of Neonatology, Clinical Research Development Center of Children′s Hospital, Hormozgan University of Medical Sciences, Bandar Abbas, Hormozgan, Iran, hums.ac.ir; ^3^ Fertility and Infertility Research Center, Hormozgan University of Medical Sciences, Bandar Abbas, Hormozgan, Iran, hums.ac.ir

**Keywords:** antihistamines, cetirizine, fetal supraventricular tachycardia, fetal tachyarrhythmia, flecainide

## Abstract

Fetal supraventricular tachycardia (SVT) represents the most common cause of sustained fetal tachyarrhythmia and may result in significant hemodynamic compromise when persistent. Data regarding the cardiovascular effects of maternal antihistamine use during pregnancy remain limited. We report a near‐term fetus with sustained SVT temporally associated with maternal ingestion of cetirizine and an antihistamine–decongestant combination for the treatment of allergic rhinitis. Owing to the persistence of the tachyarrhythmia during hospitalization, transplacental antiarrhythmic therapy with flecainide was initiated. After 48 h of treatment, successful rhythm control was achieved. Concurrent use of cetirizine and an antihistamine decongestant in a single dose during pregnancy resulted in sustained SVT in the fetus, necessitating treatment. Therefore, the use of cetirizine in the third trimester and antihistamine decongestants throughout pregnancy requires further investigation.

## 1. Introduction

Fetal arrhythmias are detected in approximately 1%–3% of pregnancies and are most often transient and benign. However, persistent fetal tachyarrhythmias may reflect significant underlying pathology and can lead to cardiac failure, hydrops fetalis, or intrauterine fetal death. Supraventricular tachycardia (SVT) is the most frequent form of sustained fetal tachycardia and is typically characterized by a regular rhythm with fetal heart rates exceeding 220 bpm [1, 2]. Advances in fetal echocardiography have enabled earlier diagnosis of fetal SVT, allowing timely therapeutic intervention to prevent hemodynamic compromise. Transplacental antiarrhythmic therapy has significantly improved fetal outcomes, particularly in nonhydropic fetuses, with low mortality rates reported when treatment is initiated promptly [2, 3]. Antihistamines are among the most frequently used medications during pregnancy. Second‐generation agents, including cetirizine, are generally considered safe with respect to teratogenic risk [4, 5]. Nevertheless, evidence regarding their potential cardiovascular effects, particularly on the fetal heart, remains limited [6]. Furthermore, the concomitant use of antihistamines and decongestants may theoretically enhance cardiovascular stimulation, potentially predisposing the fetus to tachyarrhythmias. Herein, we describe a case of sustained fetal SVT temporally associated with maternal antihistamine use in late pregnancy, underscoring a potential drug‐related risk and the importance of vigilant fetal monitoring.

## 2. Case Presentation

**Figure 1:**
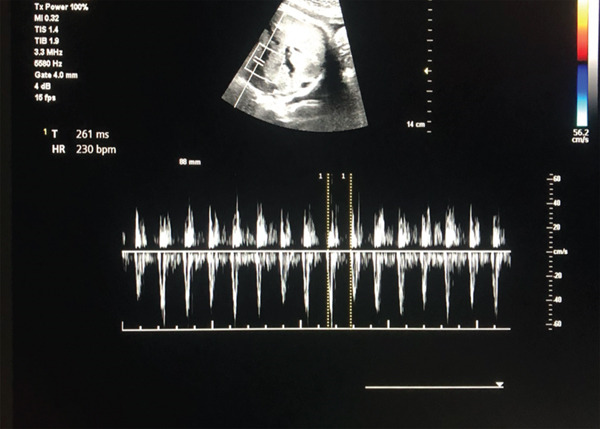
Pulse wave Doppler of aortic outflow on fetal echocardiography in a fetus at 35 weeks and 5 days of gestational age, demonstrating a heart rate of 230 bpm, at the time of admission to the hospital.

A 34‐year‐old gravida 2, para 1 woman at 35 weeks and 5 days of gestation was referred following the detection of fetal tachycardia during a routine prenatal visit with a nonstress test (NST). Her pregnancy had been uneventful, with normal fetal growth and no abnormalities on prior NST or ultrasonography. Her previous pregnancy, 5 years earlier, was uncomplicated. The patient had a history of allergic rhinitis and reported nasal congestion and sneezing without fever for 1 week before admission. Two days before admission, she had ingested a single oral dose of cetirizine 10 mg along with an antihistamine–decongestant preparation containing chlorpheniramine maleate and phenylephrine. This was her first exposure to these medications during the current pregnancy. She denied caffeine intake, herbal medication use, thyroid disease, or cardiac symptoms. Fetal movements were reported as normal, and no prior fetal heart rate abnormalities had been documented. On admission, maternal vital signs were within normal limits. Physical examination, including cardiopulmonary and otolaryngologic assessment, was unremarkable. Ultrasonography revealed a viable fetus with an estimated weight of 2.6 kg and no evidence of hydrops fetalis. Fetal echocardiography demonstrated a structurally normal heart with preserved ventricular function; however, Doppler evaluation revealed persistent SVT with a fetal heart rate of approximately 230 bpm. Maternal laboratory investigations, including complete blood count, serum electrolytes, and renal and hepatic function tests, were normal. Maternal electrocardiography and echocardiography showed no abnormalities. After 12 h of continuous fetal monitoring, repeat echocardiography confirmed persistent SVT with a fetal heart rate of 248 bpm (Figure 1).

**Figure 2:**
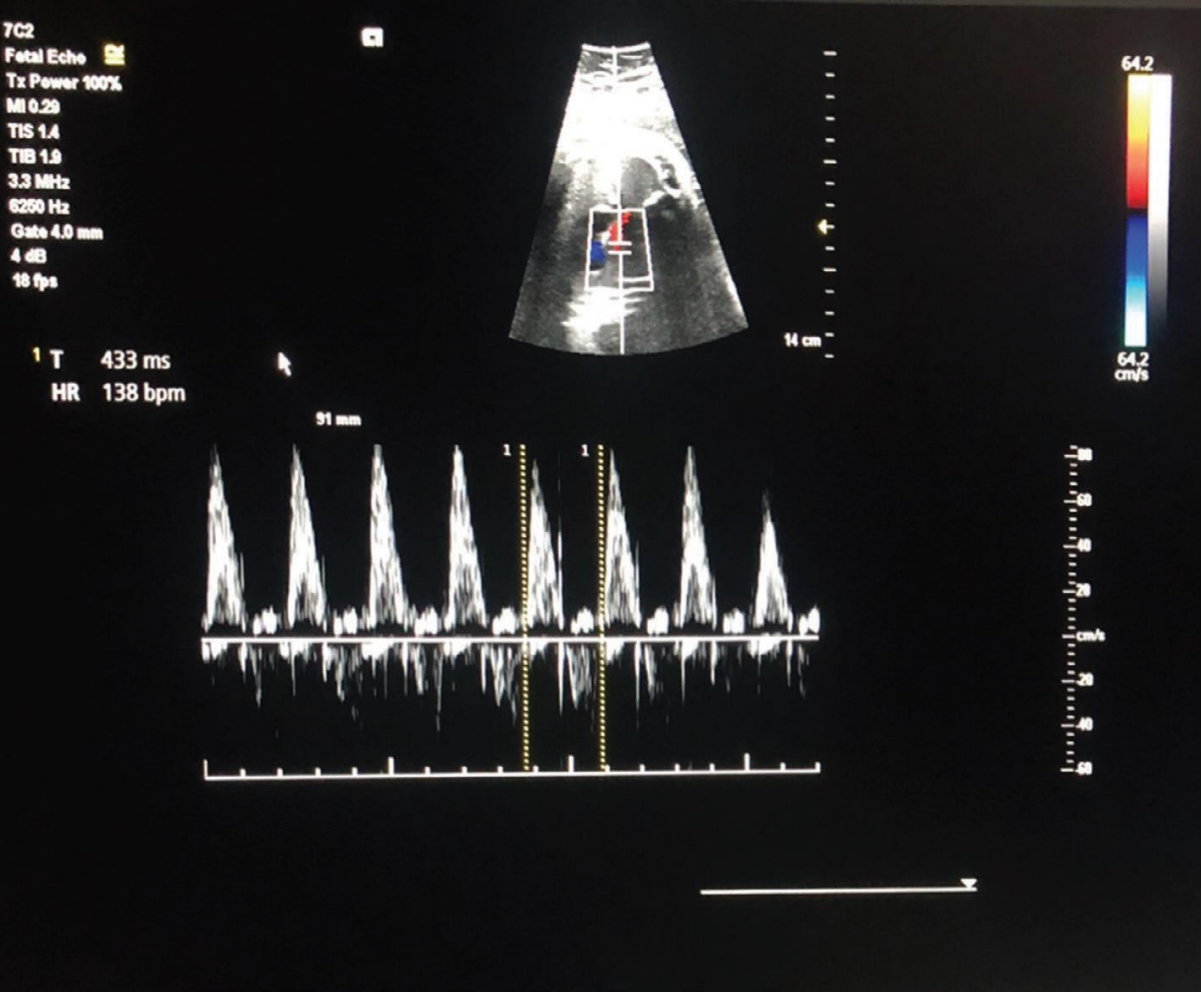
Pulse wave Doppler fetal echocardiography obtained from a five‐chamber view. The heart rate was 138 bpm after 48 h of therapy.

Following detailed counseling, the parents declined digoxin therapy; therefore, maternal oral flecainide therapy (100 mg three times daily) was initiated. Within 48 h, fetal heart rate normalized to 130–150 bpm, consistent with restoration of sinus rhythm (Figure 2).

Continuous maternal ECG monitoring revealed no conduction abnormalities or adverse effects. At 37 weeks of gestation, an elective cesarean section was performed. A female neonate weighing 2.7 kg was delivered with Apgar scores of 9 at both 1 and 5 min. Postnatal monitoring in the neonatal intensive care unit demonstrated no recurrence of SVT. Neonatal echocardiography was normal, and the infant was discharged after 5 days. At 1‐week and 3‐month follow‐up visits, the infant remained asymptomatic with a normal cardiac rhythm; longer term follow‐up data were unavailable.

## 3. Discussion

This case describes sustained fetal SVT temporally associated with maternal use of cetirizine in combination with an antihistamine–decongestant preparation in late pregnancy.

Although cetirizine is widely regarded as safe during pregnancy, most available safety data focus on teratogenicity rather than potential cardiovascular effects on the fetus. The cardiac adverse effects of cetirizine include tachycardia and QTc interval prolongation [5, 7].

Pharmacovigilance studies have suggested possible proarrhythmic signals associated with cetirizine use, although a causal relationship has not been definitively established [5].

Phenylephrine, the decongestant component, is a sympathomimetic agent with known vasoconstrictive and chronotropic properties. The concurrent administration of antihistamines and sympathomimetics may augment cardiovascular stimulation and potentially contribute to fetal tachyarrhythmia [8]. Drug‐induced fetal SVT has been described previously; for example, maternal morphine administration at 36 weeks of gestation has been reported to cause persistent fetal SVT that resolved following delivery [6], suggesting that certain medications may directly precipitate fetal arrhythmias.

Additionally, rare reports of cetirizine‐associated arrhythmias in adults, including atrial fibrillation, indicate the possibility of cardiac effects even at therapeutic doses [9].

It is also well recognized that fetal SVT can occur idiopathically in the absence of identifiable triggers. In one reported case, idiopathic fetal SVT led to hydrops fetalis and was successfully treated with transplacental sotalol and digoxin therapy [10]. These observations suggest that fetal SVT may arise from either drug‐related or idiopathic mechanisms.

In the present case, the temporal relationship between maternal antihistamine exposure and the onset of sustained fetal SVT, combined with the persistence of the arrhythmia and its resolution following antiarrhythmic therapy, supports a suspected drug‐related association.

Persistent fetal SVT lasting longer than 12 h is associated with an increased risk of ventricular dysfunction and hydrops fetalis and warrants prompt therapeutic intervention [1, 2]. Transplacental antiarrhythmic therapy remains the cornerstone of management, with flecainide demonstrating high efficacy and low mortality rates in nonhydropic fetuses [2, 3]. Flecainide is also effective in cases refractory to first‐line agents [7].

In late gestation, delivery may be considered once fetal lung maturity is achieved; however, achieving medical stabilization before delivery may reduce neonatal morbidity. In this case, successful rhythm control with flecainide before elective delivery resulted in a favorable neonatal outcome.

## 4. Conclusion

This case report describes a suspected temporal association between maternal concomitant use of cetirizine and an antihistamine–decongestant combination and the development of persistent fetal SVT. Although causality cannot be definitively established, the observed temporal relationship, persistence of the arrhythmia, and favorable response to antiarrhythmic therapy highlight the need for caution. Further studies are required to elucidate the cardiovascular effects of antihistamines, particularly when combined with decongestants, during late pregnancy.

## Author Contributions

K.R.K. was consulted, and fetal echocardiography and neonatal echocardiography were performed. K.R.K. collected the data and wrote the manuscript. S.R., N.A., and R.M.G. revised the manuscript.

## Funding

No funding was received for this manuscript.

## Disclosure

All authors have read and approved the final version of the manuscript. The corresponding author had full access to all of the data in this study and takes complete responsibility for the integrity of the data. The corresponding author affirms that this manuscript is an honest, accurate, and transparent account of the study being reported; no important aspects of the study have been omitted.

## Ethics Statement

This study was approved by the Ethics Committee of the Hormozgan University of Medical Sciences, Children′s Hospital, under the code IR.HUMS.REC. 1402.244.

## Conflicts of Interest

The authors declare no conflicts of interest.

## Supporting information


**Supporting Information** Additional supporting information can be found online in the Supporting Information section.

## Data Availability

The datasets used during the current study are available from the corresponding author upon reasonable request.
