# Supplementary material for: Unexpected Fetal Supraventricular Tachycardia Temporally Associated With Maternal Antihistamine Use: A Case Report
Source: Case Rep Obstet Gynecol. 2026 May 4;2026:6498180. doi: 10.1155/crog/6498180 (PMC13138248; doi:10.1155/crog/6498180)
Supplement: Supplementary file 1 — Supporting Information Additional supporting information can be found online in the Supporting Information section. [file CROG-2026-6498180-s001.pdf]

Hormozgan University of Medical Sciences

Research Ethics Committees Certificate

Approval ID:	IR.HUMS.REC.1402.244	Approval Date:	2023-08-13
Evaluated by:	Research Ethics Committees of Hormozgan University of Medical Sciences		
Status:	Approved		
Approval Statement:	The project was found to be in accordance to the ethical principles and the national norms and standards for conducting Medical Research in Iran.<br>Notice:<br>1. Although the proposal has been approved by the Biomedical Research Ethics Committee, meeting the professional and legal requirements is the sole responsibility of the PI and other project collaborators.<br>2. This certificate is reliant on the proposal/documents received by this committee on 2023-08-13. The committee must be notified by the PI as soon as the proposal/documents are modified.		
Proposal Title:	Antihistamine induced fetal supraventricular tachycardia: A case report		
Principal Investigator:	Name: KHADIJEH RIAZI KERMANI<br>Email: riazikermani2014@gmail.com		

Dr. Gholamali Javdan
Committee Director
Hormozgan University of Medical Sciences

Dr. vali alipour
Committee Secretary
Hormozgan University of Medical Sciences
